# Asymmetric Electrokinetic Energy Conversion in Slip Conical Nanopores

**DOI:** 10.3390/nano12071100

**Published:** 2022-03-27

**Authors:** Chih-Chang Chang

**Affiliations:** Department of Industrial Technology Education, National Kaohsiung Normal University, Kaohsiung 824, Taiwan; ccchang0803@gmail.com; Tel.: +886-7-7172930 (ext. 7622)

**Keywords:** electrokinetics, nanofluidics, electric double layer, electrokinetic energy conversion, ion concentration polarization, hydrodynamic slippage, conical nanopore

## Abstract

Ion current rectification (ICR) phenomena in asymmetric nanofluidic structures, such as conical-shaped nanopores and funnel-shaped nanochannels, have been widely investigated in recent decades. To date, the effect of asymmetric nanofluidic structures on electrokinetic power generation driven by the streaming current/potential has not been explored. Accordingly, this study employed a numerical model based on the Poisson equation, Nernst–Planck equation, and Navier–Stokes equation to investigate the electrokinetic energy conversion (EKEC) in a conical nanopore while considering hydrodynamic slippage. The results indicated that the asymmetric characteristics of streaming current (short-circuit current), streaming potential (open-circuit voltage), maximum power generation, maximum conversion efficiency, and flow rate were observed in conical nanopores under the forward pressure bias (tip-to-base direction) and reverse pressure bias (base-to-tip direction) once the nonequilibrium ion concentration polarization (ICP) became considerable. The rectification behaviors in the streaming current, maximum power, and maximum conversion efficiency were all shown to be opposite to those of the well-known ICR in conical nanopores. In other words, the reverse pressure bias revealed a higher EKEC performance than the forward pressure bias. It was concluded that the asymmetric behavior in EKEC is attributed to the asymmetric electrical resistance resulting from asymmetric ion depletion and ion enrichment. Particularly, it was found that the decrease in electrical resistance (i.e., the change in electrical resistance dominated by the ion enrichment) observed in the reverse pressure bias enhanced the maximum power and maximum conversion efficiency. The asymmetric EKEC characteristics became more significant with increasing slip length, surface charge density, cone angle, and pressure bias, especially at lower salt concentrations. The present findings provide useful information for the future development of EKEC in engineered membranes with asymmetric nanopores.

## 1. Introduction

With the fast-growing demand for electricity worldwide and electricity production based on fossil fuels, the development of electricity from new, clean, and sustainable energy sources has become one of the most crucial tasks in the fields of engineering and science. Among various renewable energy sources that have been proposed, “hydrovoltaics” [1,2], namely, electricity generation from water, has attracted great attention recently since water covers about 71% of Earth’s surface and absorbs about 35% of solar radiation received by Earth, corresponding to a huge power of 6 × 10^16^ W [3]. The absorbed energy drives water evaporation and gives rise to moisture, rainfall, snowfall, rivers, lake, etc., establishing a global water cycle. In other words, water is the largest energy reservoir, regulator, and balancer on Earth. Most of Earth’s surface water is in oceans, rivers, lakes, etc., and contains tremendous energy in a variety of forms, such as kinetic energy, evaporation energy [4,5], and salinity-gradient energy [6,7]. Unlike the well-known hydroelectric power plant that converts the kinetic energy of flowing water transformed from the potential energy stored in a water reservoir into electricity through a water turbine and generator, hydrovoltaics refers to the direct generation of electricity from water by water-solid interfacial phenomena, e.g., the electrokinetic effect.

One hundred and sixty-three years ago, German physicist G. Quincke first observed that a voltage can be generated when an aqueous (electrolyte) solution is forced through a narrow channel under a hydrostatic pressure gradient [8]. This electrification phenomenon was attributed to the presence of a nonelectroneutral electric double layer (EDL) at the water-solid interface. The excess counterions within the EDL are carried toward the downstream end and result in a convective current, which is usually termed the streaming current, i.e., short-circuit current. Corresponding to the streaming current, there is an open-circuit voltage termed the streaming potential. The energy harvesting from the streaming current/potential generated by the flowing water in charged channels is termed electrokinetic energy conversion (EKEC) or electrokinetic power generation. The research of Osterle and Morrison [9,10] may be viewed as pioneering studies of EKEC. In their studies, low conversion efficiency ranging from 0.32% to 0.9% was predicted. Thanks to advancements in nanotechnology and materials science, EKEC has received renewed attention in the field of nanofluidics in recent decades [11,12,13,14,15,16,17]. The results indicate that the EKEC efficiency can be increased when pressure-driven flow occurs through nanofluidic channels in the presence of an overlapped EDL, i.e., the channel has a higher ion selectivity. For an electrolyte consisting of simple monovalent ions such as Li^+^, theoretical maximum EKEC efficiencies of approximately 11.6% and 9.7% were predicted in negatively charged nanochannels and nanopores [12,13], respectively, but the highest EKEC efficiency that has been measured in experiments to date has not surpassed 5% in engineered nanochannels and nanopores [14,15]. In other words, the low conversion efficiency leaves a large room for improvement in electrokinetic power generation. In general, the no-slip velocity condition on the solid surface is a good approximation for most fluid mechanics issues. However, more recent studies have observed that the phenomenon of liquid slip may occur on solid surfaces, especially hydrophobic surfaces. In micro/nanoscale liquid flows, experiments and MD simulations have provided evidence of slip lengths ranging from several nanometers to micrometers [18,19,20,21,22,23], which is very comparable with the channel scales. For example, a slip length as large as 33 μm was observed when the liquid flow was forced through carbon nanotube filters [18]. Bouzigues et al. reported that electroosmotic flow (EOF) over an octadecyl-trichloro-silane (OTS) surface has a slip length of approximately 36 nm [19]. In addition, Xie et al. reported that the slip length is approximately 16 nm in graphene-covered silica nanochannels [23]. It is known that a larger slip length results in a larger slip flow velocity in nanochannels. This implies that the water/ion transport in nanochannels can be greatly enhanced when the slip effect becomes significant. Hence, the pressure-driven streaming current/potential is expected to be amplified in slip nanochannels. As a result, hydrodynamic slippage was proposed to improve the EKEC efficiency in nanochannels and nanopores to a practical efficiency [13,24,25]. For ion-selective nanopores, Chang and Yang reported that the EKEC efficiency can be greatly improved to 40% when the ratio of slip length to radius is larger than 0.7 [13].

In recent decades, unique transport phenomena due to the presence of asymmetric EDL distribution in asymmetrically shaped nanofluidic structures have received considerable research interest [26,27,28,29,30]. Asymmetric nanofluidic structures have been proposed, including polymer nanopores, silicon nanopores, mica nanopores, gold nanotubes, glass nanocapillaries, and glass nanofunnels [31,32]. The occurrence of unique transport phenomena is mainly attributed to the presence of ion concentration polarization (ICP), including ion depletion and enrichment inside the nanofluidic structures. One of the unique transport phenomena observed in these nanofluidic structures is known as ion current rectification (ICR), which is analogous to the current rectification observed in solid-state diode rectifiers. In conical nanopores, ICR is largely dependent on the polarity of voltage bias and the polarity of surface charge. When the base of the conical nanopore is grounded and the positive voltage bias is applied on the tip side, the negatively charged conical nanopore exhibits a higher ionic current and conductance due to the occurrence of ion enrichment inside the nanopore. Conversely, when the negative voltage bias is applied on the tip side, the negatively charged conical nanopore exhibits a lower ionic current and conductance due to the occurrence of ion depletion inside the nanopore. Furthermore, the ICR phenomenon was used to develop a nanofluidic diode [33] that has been widely applied in biochemical sensing [29,30,34]. Regarding the fundamental studies of the ICR phenomenon, Hsu et al. recently showed that EOF, the type of salt, salt concentration, and cone angle all influence the ICR in conical nanopores [35,36]. It was found that a larger ICR ratio is obtained at a higher voltage bias if EOF is taken into account. Lin et al. reported that the ICR phenomenon can also be observed in highly charged PET conical mesopores with a tip diameter of 400 nm at a high pH of 11 (surface charge density of approximately −160 C/m^2^), even though the EDL does not overlap inside the pores [37]. In the presence of a salinity gradient, Lin et al. found that the current-voltage curve revealed a negative differential resistance in a conical mesopore due to the synergistic effect of EOF and diffusioosmotic flow [38]. More recently, Ma et al. reported that the ICR of ultrashort-length conical nanopores could be tuned by controlling individual charged surfaces, including the inner pore surface and exterior pore surfaces on the tip and base side [39]. The results showed that ICR ratios from 2 to 170 could be achieved by different configurations of charged surfaces.

In addition to the ICR phenomenon, the EOF rectification phenomenon due to the occurrence of the ICP was later observed theoretically and experimentally in asymmetric nanofluidic structures [28,40,41,42,43,44], and plays an important role in developing electroosmotic pumps based on engineered membranes or nanochannels with asymmetric geometry [41,45,46]. It was found that the ICR and EOF rectification phenomena typically occur simultaneously at the low salt concentrations when the voltage bias is applied through an asymmetric nanofluidic structure. The EOF rectification reveals a contrasting rectification behavior to that of the well-studied ICR phenomenon. For example, Yeh et al. [41] reported that the negatively charged conical nanopore revealed a higher EO flow rate and lower current when the voltage bias was applied from the base side to the tip side, whereas it revealed a lower EO flow rate and higher current when the voltage bias was applied from the tip side to the base side. This unique phenomenon results in a higher pumping pressure and pumping efficiency when the voltage bias is applied from the base side to the tip side [41]. In an application of asymmetrically shaped pores to osmotic energy harvesting, Yeh et al. reported that the conical nanopore revealed a higher ion selectivity when the salinity gradient was from the base side to the tip side, which resulted in a higher power and conversion efficiency [47]. Lin et al. recently reported that the osmotic power density could be improved to approximately 950 W/m^2^ by utilizing a conical mesopore with the tip region filled with the polyelectrolyte poly-L-lysine [48].

**Figure 1:**
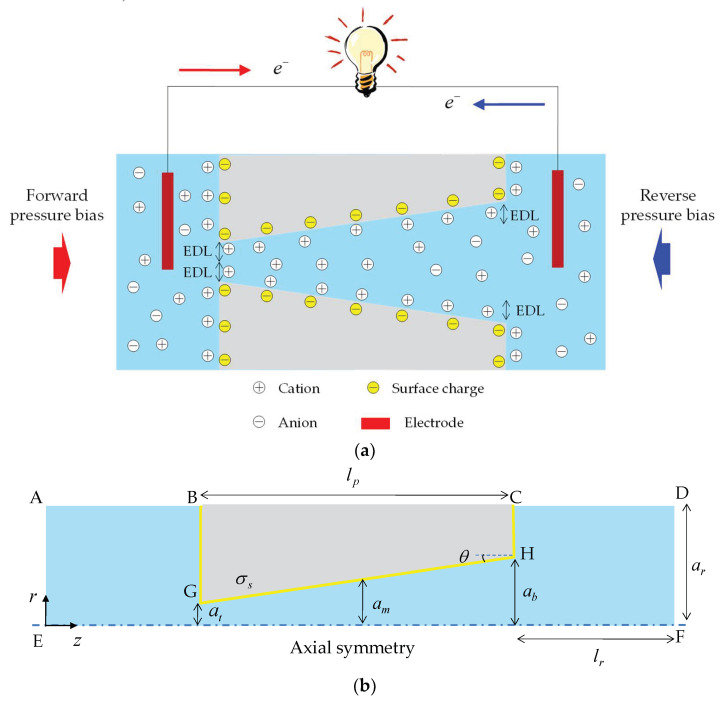
(**a**) Schematic representation of electrokinetic power generation under a forward/reverse pressure bias in a conical nanopore bearing negative surface charges. (**b**) Schematic illustration of 2D axisymmetric geometry of a conical nanopore used in the computation.

In light of the slip-enhanced water/ion transport and asymmetrical-shape-induced unique transport phenomena at the nanoscale mentioned above, this study aimed to investigate the EKEC in conically shaped nanopores considering hydrodynamic slippage through parametric numerical simulations. To the best of my knowledge, this issue has not yet been discussed. As depicted in Figure 1a, the electrokinetic power generation is driven by applying pressure bias through a negatively charged conical nanopore. The forward pressure bias refers to the pressure-driven flow in the tip-to-base direction, whereas the reverse pressure bias refers to that in the opposite direction. Adopting a continuum model, which is a set of the Navier-Stokes equation and Poisson-Nernst–Planck equations, the influences of the pressure bias, salt concentration, slip length, surface charge density, and cone angle on the electrokinetic power generation and energy conversion efficiency of conical nanopores were examined in this study. As expected, the asymmetric behavior of electrokinetic power generation due to the occurrence of ICP was observed. Notably, the EKEC performance was enhanced when ion enrichment played a dominant role in the reverse pressure bias.

## 2. Mathematical Model

In this study, a conical nanopore of length *l*_p_, tip radius a_t_, base radius a_b_, and half-cone angle *θ* connected with two identical large reservoirs of length *l*_r_ and radius a_r_ was considered, as shown in Figure 1b, where a_m_ is the radius at the middle of the conical nanopore and *σ*_s_ is the constant surface charge density of the pore surface, respectively. The geometric scales of *l*_p_ = 2 μm, a_t_ = 10 nm, and *l*_r_ = a_r_ = 2 μm were specified in this study. The half-cone angle of *θ* depends on the base radius a_b_. The governing equations based on the continuum assumption and the boundary conditions adopted in the simulation are described below in detail.

### 2.1. Governing Equations

For dilute solutions, the steady-state ion transport through the conical nanopore arising from electromigration, diffusion, and convection is described by the Nernst–Planck equation without considering the chemical reaction.

(1) $$j_{i}=-\frac{Fz_{i}D_{i}}{R_{u}T}\nabla \phi -D_{i}\nabla c_{i}+c_{i}u$$

(2) $$\nabla \cdot j_{i}=0$$

where ∇ is the Del operator in cylindrical coordinates and the subscript *i* denotes the *i*th ion species. $j_{i}$, $c_{i}$, $D_{i}$, and $z_{i}$ represent the molar flux, molar concentration, diffusivity, and valence of the *i*th ion species, respectively. u, ϕ, F, $R_{u}$, and T are the fluid velocity, electric potential, Faraday constant, universal gas constant, and temperature, respectively.

The ion current (*I*) through the conical nanopore can be calculated by

(3) $$I=\int_{A}F\sum_{i}z_{i}j_{i}\cdot ndA$$

where *A* is the cross-sectional area of the conical nanopore and **n** denotes the unit normal vector to *A*.

According to electrostatics, the electric potential is described by the Poisson equation

(4) $$\nabla^{2}\phi =-\frac{\rho_{e}}{\varepsilon_{f}\varepsilon_{0}}$$

where the net charge density is represented by $\rho_{e}=F\sum_{i}z_{i}c_{i}$. $\varepsilon_{f}$ and $\varepsilon_{0}$ are the dielectric constant of fluid and the permittivity of vacuum, respectively.

Adopting the continuum assumption [49], the incompressible steady-state creeping flow (i.e., Reynolds number much smaller than unity) through the conical nanopore is described by the continuity equation and the Navier–Stokes equation, including the electric body force term (i.e., Coulombic force, $\rho_{e}E$)

(5) ∇·u=0

(6) $$0=-\nabla p+\mu \nabla^{2}u+\rho_{e}E$$

where μ and p are the fluid viscosity and pressure, respectively. The electric field is represented by E=−∇ϕ.

The coupling of Equations (1), (4)–(6), termed the Poisson-Nernst–Planck + Navier–Stokes (PNP + NS) model, was solved to describe ion transport through the conical nanopores.

### 2.2. Boundary Conditions

To specify the boundary conditions associated with the PNP + NS model mentioned above, this study assumed that the nanopore surfaces were ion- and fluid-impermeable and had a constant surface charge density *σ*_s_. Additionally, the well-known Navier slip velocity on the surface of the nanopore, $u=bn\cdot \left(\nabla u+{\left(\nabla u\right)}^{T}\right)$, was adopted to describe the hydrodynamic slippage, where b is referred to as the slip length and n denotes the normal to the surface directed into the fluid [50]. Referring to the geometry of the conical nanopore illustrated in Figure 1b, the boundary conditions used in the computation are summarized in Table 1.

## 3. Results and Discussion

In this study, the PNP + NS model associated with the boundary conditions listed in Table 1 was solved by the finite element method-based commercial software COMSOL Multiphysics (COMSOL Inc., Stockholm, Sweden.). The KCl salt solution was considered the working fluid through the conical nanopore. Assuming that the salt concentration was much higher than the concentrations of H^+^ and OH^−^ in the neutral aqueous solution (i.e., pH = 7), the cation and anion fluxes through the conical nanopore were dominated by K^+^ and Cl^−^ ions, respectively, and the flux contributions from H^+^ and OH^−^ were both neglected. The parameter values used in the computation were $R_{u}=8.314\;J/\mathrm{mol}\cdot K$, F=96485 C/mol, T=298 K, $D_{K^{+}}=1.96\times 10^{-9}{\;m}^{2}/s$, $D_{{\mathrm{Cl}}^{-}}=2.02\times 10^{-9}{\;m}^{2}/s$, $\varepsilon_{f}=78.5$, $\varepsilon_{0}=8.854\times 10^{-12}\;F/m$, and $\mu =8.9\times 10^{-4}\;\mathrm{Pa}\cdot s$. Previously, Ren and Stein [24] theoretically evaluated that slip flow enhanced EKEC efficiency in nanofluidic structures by considering the slip length in the range between 0 and 50 nm. The calculated results revealed that the practical efficiency is expected for the moderate slip length of 20–30 nm. Accordingly, a moderate slip length of b = 20 nm was assumed to conduct the parametric numerical study of EKEC characteristics in conical nanopores.

### 3.1. Mesh Independence Study

**Figure 2:**
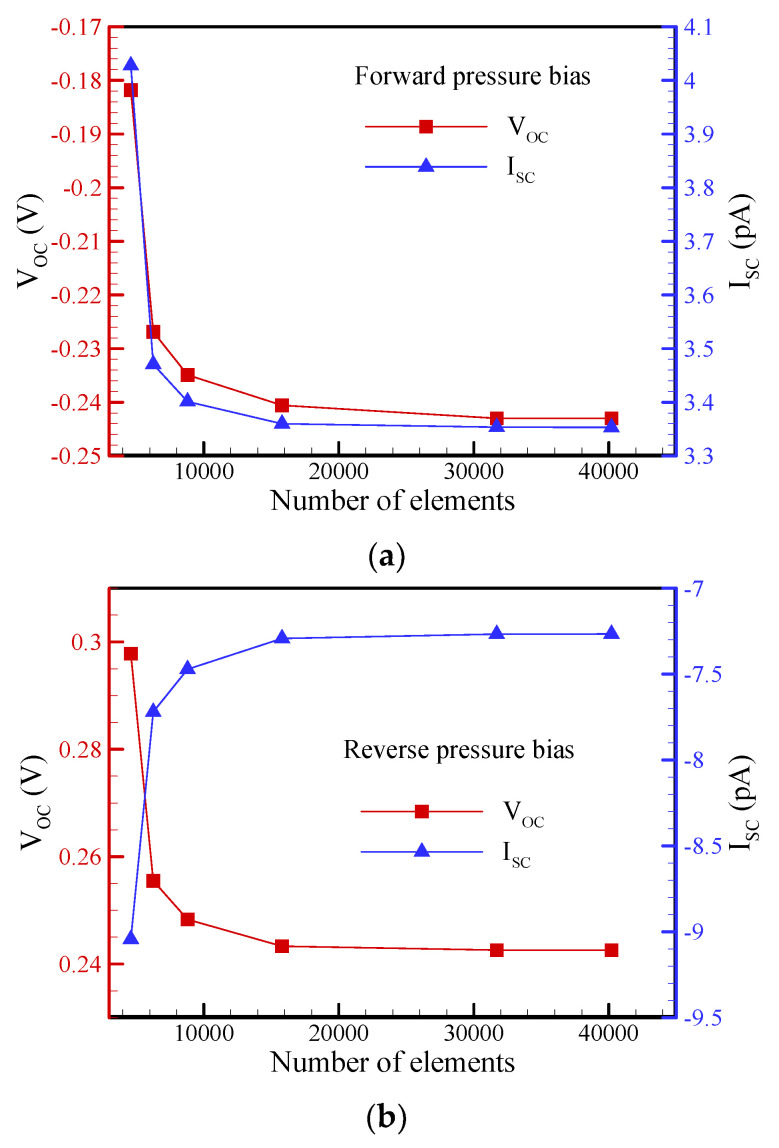
The dependence of the number of mesh elements on the numerical solutions of the open-circuit voltage (V_OC_) and the short-circuit current (I_SC_) through a conical nanopore: (**a**) forward pressure bias (tip-to-base); (**b**) reverse pressure bias (base-to-tip).

The computations were performed using quadrilateral elements and a refined mesh. In other words, a finer mesh was used near the charged surfaces, the entrance, and the exit of the conical nanopore to resolve the subtle changes in physical variables in the PNP + NS model. The solution independence of the mesh size was carefully studied before reporting the final calculated results. The results presented in this study were all obtained using a mesh containing 31,700~58,000 elements. Figure 2 shows an example of a mesh independence test, where the conical nanopore had a base radius of a_b_= 50 nm, a half-cone angle of *θ* = 1.2°, a surface charge density of *σ*_s_ = −5 mC/m^2^, and a slip length of b = 20 nm. Under a pressure bias of p_ext_ = 250 kPa, the mesh dependence on the open-circuit voltage (V_OC_) and short-circuit current (I_SC_) of the electrokinetic power generation for forward/reverse bias were examined at a salt concentration of 0.1 mM. Note that the open-circuit voltage refers to the streaming potential, while the short-circuit current refers to the streaming current. It can clearly be seen that mesh-independent solutions were obtained when the mesh comprised 31,700 elements.

### 3.2. Pressure Bias Dependence on the Open-Circuit Voltage (V_OC_), Short-Circuit Current (I_SC_), and Flow Rate (Q) of EKEC

**Figure 3:**
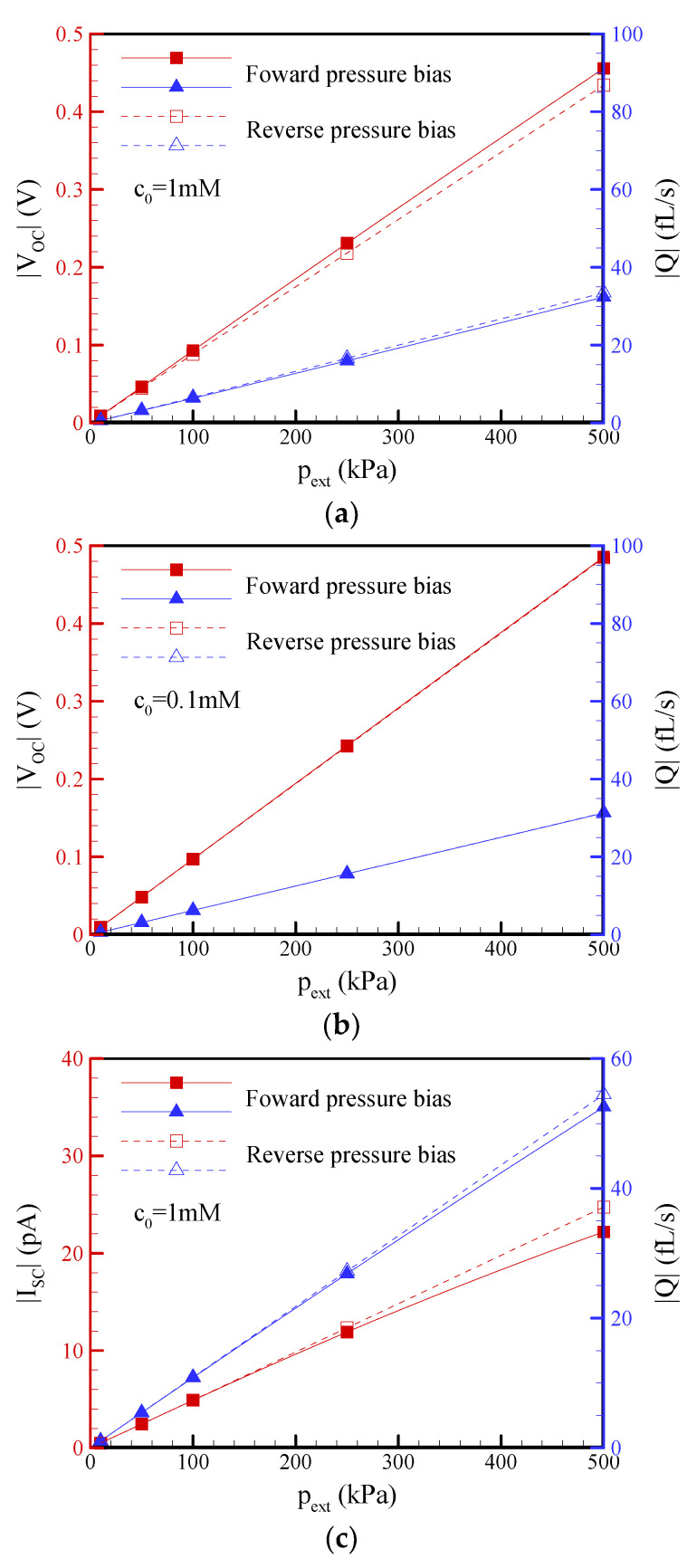

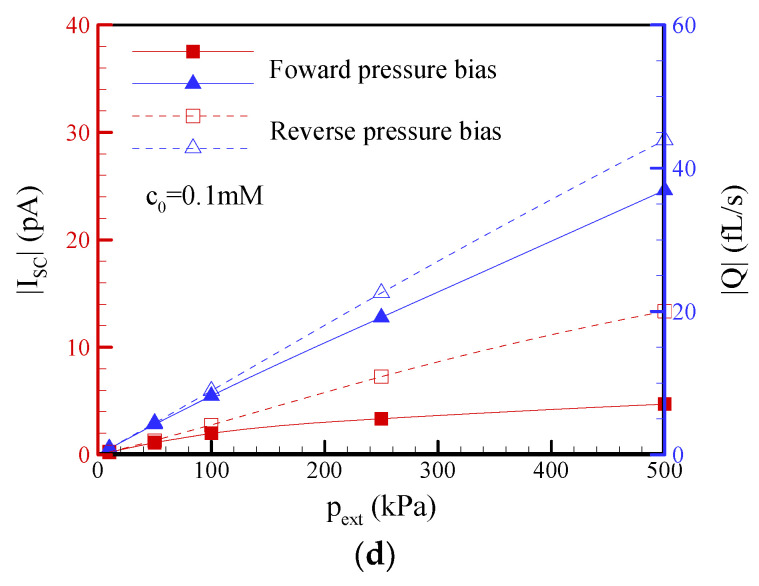
The dependence of the pressure bias on the open circuit voltage (V_OC_), short circuit current (I_SC_), and flow rate (Q) through a conical nanopore at salt concentrations of (**a**,**c**) c_0_ = 1 mM; (**b**,**d**) c_0_ = 0.1 mM. Note that *θ* = 1.2°, *σ*_s_ = −5 mC/m^2^, and b = 20 nm.

**Figure 4:**
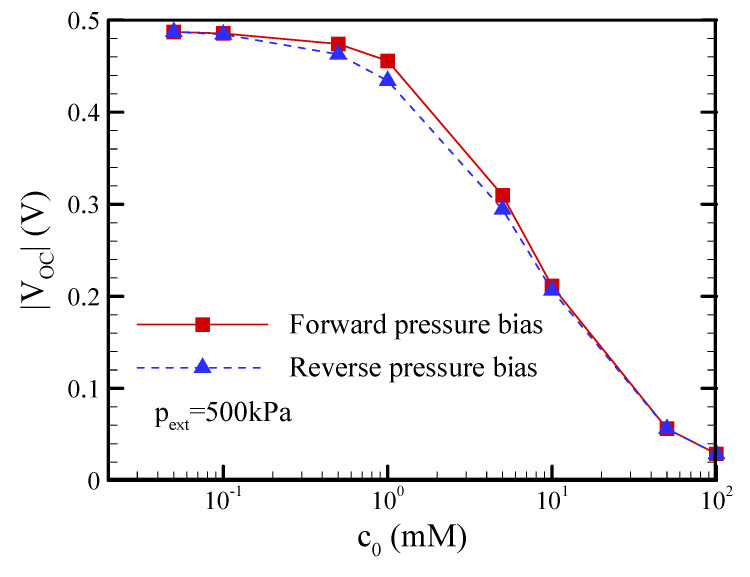
The dependence of the salt concentration on the streaming potential rectification in a conical nanopore. Note that *θ* = 1.2°, *σ*_s_ = −5 mC/m^2^, and b = 20 nm.

In this section, a conical nanopore with a half-cone angle of *θ* = 1.2°, a surface charge density of *σ*_s_ = −5 mC/m^2^, and a slip length of b = 20 nm is specified. Figure 3 shows comparisons of the open-circuit voltage, short-circuit current, and flow rate (Q) between the forward and reverse pressure biases at different salt concentrations of 0.1 mM and 1 mM. Under the open-circuit condition, Figure 3a,b reveal the linear characteristics of the V_OC_-p_ext_ and Q-p_ex_ curves since nonequilibrium ICP phenomena did not occur when the net ionic current was zero. In addition, Figure 3a shows that the open-circuit voltage in the forward pressure bias was slightly larger than that in the reverse pressure bias. In other words, streaming potential rectification was observed at a salt concentration of 1 mM, while it disappeared at a lower salt concentration of 0.1 mM. Interestingly, Figure 4 reveals that the streaming potential rectification appeared in the range between the salt concentrations of 0.1 mM and 10 mM. It was inferred that the streaming potential rectification may be due to the asymmetric behavior of axial ion distribution under the forward and reverse pressure biases when EDL overlapped on the tip side while it did not overlap on the base side in this salt concentration regime. When EDL overlapped either on the tip side or the base side of a conical nanopore at a lower salt concentration, it was found that the asymmetric behavior of axial ion distribution disappeared. As a result, the streaming potential rectification was not observed in the lower salt concentration regime. On the other hand, the streaming potential rectification also disappeared when EDLs on the tip and base sides both did not overlap at a higher salt concentration. This streaming potential rectification resulted in a very small flow rectification due to the electroviscous effect arising from the streaming potential-driven electroosmotic flow in the opposite direction of pressure-driven flow [51]. In contrast, the I_SC_-p_ext_ and Q-p_ex_ curves presented in Figure 3c,d reveal obvious nonlinear behaviors due to the occurrence of nonequilibrium ICP phenomena under the short-circuit condition. In the reverse pressure bias, the conical nanopore had a higher streaming current and flow rate than that in the forward pressure bias, i.e., significant streaming current rectification and flow rectification in a conical nanopore were observed, especially at a lower salt concentration of 0.1 mM. The rectification phenomena became more significant as the pressure bias increased.

**Figure 5:**
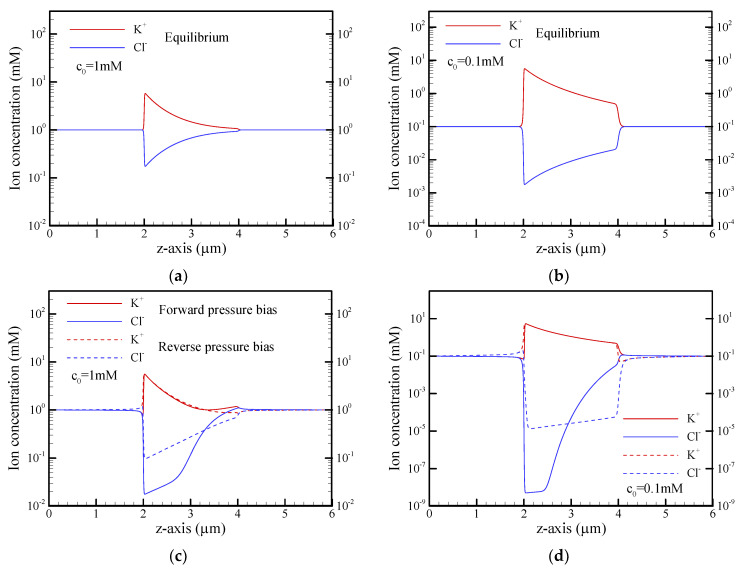
Plots of the cation (K^+^) and anion (Cl^−^) concentrations along the centerline of the reservoir and conical nanopore for salt concentrations of c_0_ = 1 mM and c_0_ = 0.1 mM under the short-circuit condition and different pressure biases. (**a**,**b**) p_ext_ = 0 kPa (equilibrium state); (**c**,**d**) p_ext_ = 500 kPa. Note that the solid and dashed lines in (**c**,**d**) represent the results of forward pressure bias and reverse pressure bias, respectively. Note that *θ* = 1.2°, *σ*_s_ = −5 mC/m^2^, and b = 20 nm.

**Figure 6:**
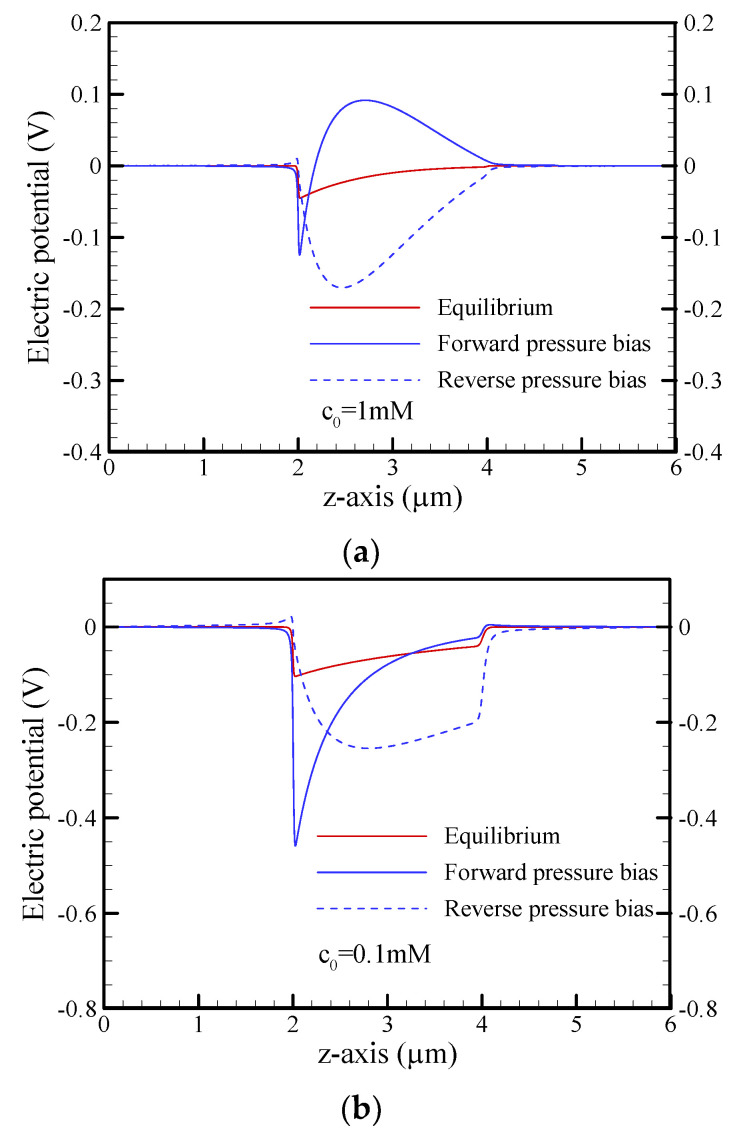
Plots of the electric potential along the centerline of the conical nanopore under the short-circuit condition and a pressure bias of p_ext_ = 500 kPa for different salt concentrations of (**a**) c_0_ = 1 mM and (**b**) c_0_ = 0.1 mM. Note that *θ* = 1.2°, *σ*_s_ = −5 mC/m^2^, and b = 20 nm.

Figure 5 shows the distributions of cation (K^+^) and anion (Cl^−^) concentrations along the centerline of the conical nanopore under different operating conditions. At equilibrium, the ion distributions for salt concentrations of c_0_ = 1 mM and c_0_ = 0.1 mM are shown in Figure 5a,b, respectively. As expected, the cation concentration was much larger than the anion concentration on the tip side due to the presence of the strongly overlapped EDL at a salt concentration of c_0_ = 0.1 mM (a_t_/*λ*_D_ = 0.3), while this phenomenon gradually became insignificant on the base side (a_b_/*λ*_D_ = 1.5), especially at the higher salt concentration of c_0_ = 1 mM (a_t_/*λ*_D_ = 1.0 and a_b_/*λ*_D_ = 5). Note that a/*λ*_D_ is the dimensionless Debye-Hückel parameter and *λ*_D_ is the Debye length, i.e., the characteristic thickness of the EDL. When a pressure bias of 500 kPa was applied under the short-circuit condition, it can clearly be seen that the ion distributions deviated from the equilibrium state due to the presence of the nonequilibrium ICP phenomenon, as shown in Figure 5c,d, especially in the case of c_0_ = 0.1 mM. In the forward pressure bias, ion depletion occurred around the tip of the nanopore, while ion enrichment was observed around the base of the nanopore. In contrast, ion depletion (ion enrichment) occurred around the base (tip) of the nanopore in the reverse pressure bias. The anion depletion inside the conical nanopore was obvious in the forward pressure bias and in the reverse-pressure bias (it was more significant in the forward pressure bias), and the anion concentration around the tip approached zero, as shown in Figure 5d. This ultralow concentration resulted in a higher local electrical resistance. As shown in Figure 6, the electric potential distribution inside the conical nanopore revealed a stronger electric field in the opposite direction of the forward pressure bias, which simultaneously drove an ion current and an EOF in the opposite direction of the pressure-driven flow. This resulted in a significant reduction in the short-circuit current and flow rate in the forward pressure bias, as shown in Figure 3c,d, especially at the salt concentration of c_0_ = 0.1 mM.

### 3.3. Pressure Bias Dependence on the I-V Characteristic Curve, Maximum Power, and Maximum Conversion Efficiency of EKEC

**Figure 7:**
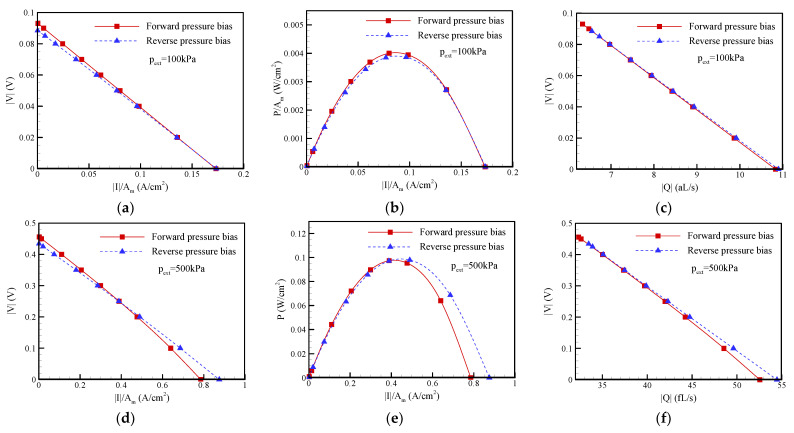
Plots of the I-V curve, I-P curve, and Q-V curve for EKEC in a conical nanopore at a salt concentration of c_0_ = 1 mM under different pressure biases: (**a**–**c**) p_ext_ = 100 kPa; (**d**–**f**) p_ext_ = 500 kPa. Note that *θ* = 1.2°, *σ*_s_ = −5 mC/m^2^, and b = 20 nm. A_m_ = π (a_m_)^2^, where a_m_ is the radius at the middle of the conical nanopore.

**Figure 8:**
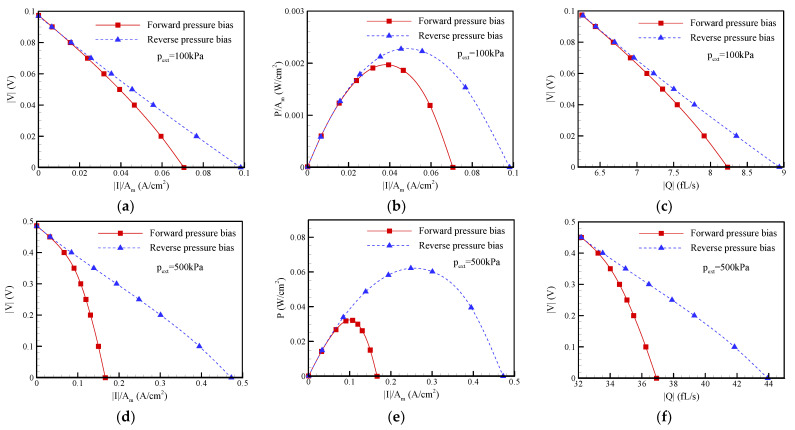
Plots of the I-V curve, I-P curve, and Q-V curve for EKEC in a conical nanopore at a salt concentration of c_0_ = 0.1 mM under different pressure biases: (**a**–**c**) p_ext_ = 100 kPa; (**d**–**f**) p_ext_ = 500 kPa. Note that *θ* = 1.2°, *σ*_s_ = −5 mC/m^2^, and b = 20 nm. A_m_ = π (a_m_)^2^, where a_m_ is the radius at the middle of the conical nanopore.

As discussed in the last section, it is believed that the ion current and flow rectification phenomena are due to the occurrence of ICP impact the EKEC in the conical nanopore. Figure 7 and Figure 8 show the current-voltage (I-V) curve, current-power (I-P) curve, and flow rate-voltage (Q-V) curve of EKEC for salt concentrations of c_0_ = 1 mM and c_0_ = 0.1 mM, respectively, under different pressure biases. It should be noted that the voltage |V| is controlled by adjusting the resistance of external load (R_load_) in practice [12,13,14]. When R_load_ is much larger than the electrical resistance of conical nanopore (R), i.e., R_load_ >> R, no current passes through the external load and the EKEC system reveals an open circuit, i.e., |V| = |V_OC_| and |I| = 0. When R_load_ is gradually decreased, the current (|I|) passing through the external load increases and the voltage |V| decreases. Once R_load_ << R, the EKEC system reveals a short circuit, i.e., |V| = 0 and |I| = |I_SC_|. In Figure 7, it can be seen that the I-V and Q-V curves both revealed linear behavior either in the forward-pressure bias or the reverse-pressure bias under a lower pressure bias of 100 kPa. The maximum power generated in the forward-pressure bias was slightly larger than that in the reverse-pressure bias since the former has a slightly larger open-circuit voltage. When a higher pressure bias was applied, weakly nonlinear I-V and Q-V curves could be observed in the forward pressure bias, as presented in Figure 7d,f, because the ICP phenomenon became significant as the pressure bias increased. The conical nanopore exhibited a higher electrical resistance in the forward-pressure bias. As a result, the maximum power (P_max_) generated in the reverse pressure bias became larger than that in the forward pressure bias, as revealed in Figure 7e. Note that the power density (P/A_m_) shown in I-P curve is the product of the current density (|I|/A_m_) and voltage (|V|) shown in I-V curve. It can clearly be seen that the power density was zero under the open-circuit condition (i.e., |V| = |V_OC_| and |I| = 0). The power density increased with decreasing the voltage |V| (i.e., decreasing the external load resistance R_load_). After reaching a maximum value, the power density began decreasing with decreasing the voltage |V|. Under the short-circuit condition, the power density decreased to zero since |V| = 0 and |I| = |I_SC_|. As a result, a parabolic I-P curve was obtained. The maximum power refers to the maximum value of the power in I-P curves. In equilibrium EKEC, it was found that the maximum power follows the relationship of P_max_ = |I_SC_||V_OC_|/4 [12,13], but it may become invalid once the nonequilibrium ICP phenomenon occurs. When the EDL overlap became more significant at the lower salt concentration of c_0_ = 0.1 mM, the I-V and Q-V curves presented in Figure 8 exhibited significant nonlinear relationships as a result of the strong ICP effect illustrated in Figure 5 and Figure 6, especially for the forward-pressure bias. As mentioned in the last section, a considerable reduction in the short circuit current was observed when the forward-pressure bias was applied. The electrical resistance of the conical nanopore significantly increased in the forward bias, and the maximum power dramatically decreased. The reverse pressure bias revealed a higher maximum power than the forward pressure bias. In addition, the ICP phenomenon also resulted in a decrease in the flow rate, as shown in Figure 8c,f, which may have an influence on the input mechanical power (|Q|p_ext_).

**Figure 9:**
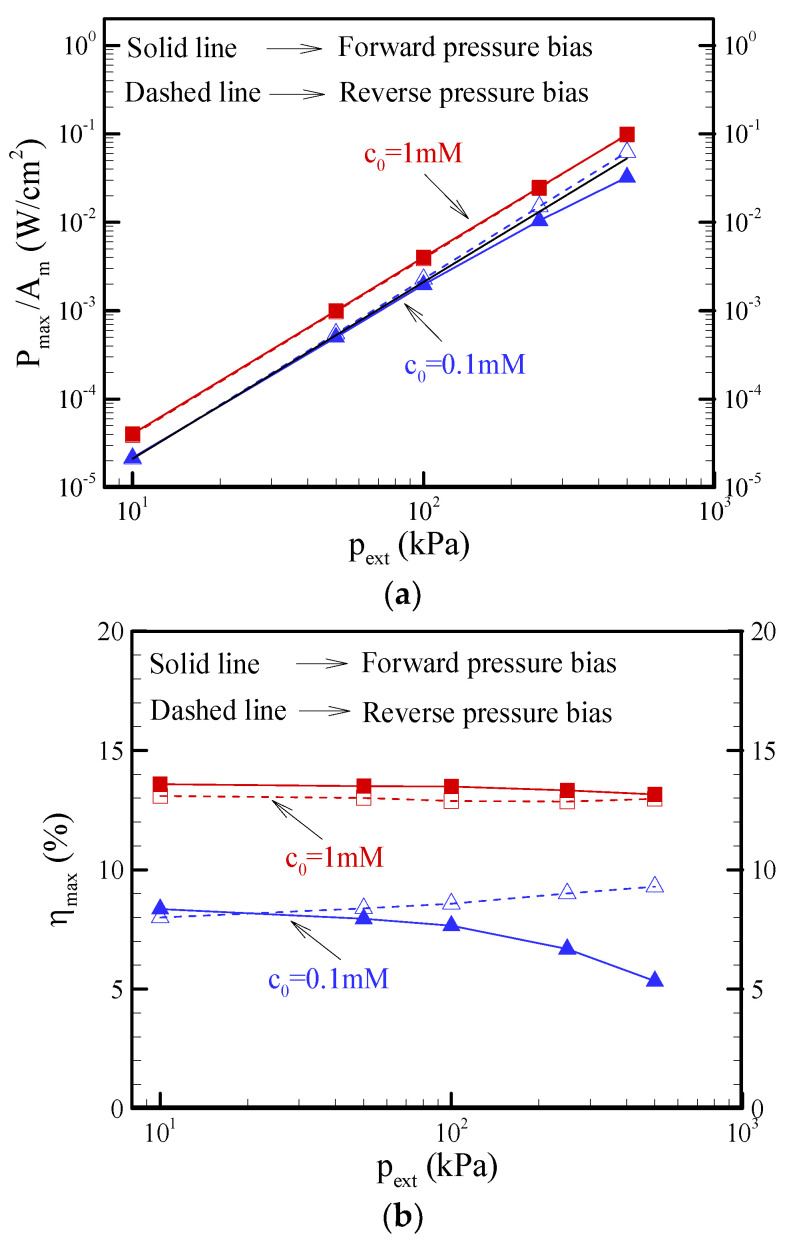
Plots of the (**a**) maximum power density (P_max_/A_m_) and (**b**) maximum conversion efficiency (*η*_max_) as a function of the pressure bias at the different salt concentrations. Note that *θ* = 1.2°, *σ*_s_ = −5 mC/m^2^, and b = 20 nm. A_m_ = π (a_m_)^2^, where a_m_ is the radius at the middle of the conical nanopore. The black solid line in (a) refers to the maximum power density predicted for the salt concentration of c_0_ = 0.1 mM under the equilibrium EKEC assumption.

Figure 9a reveals the variations in the maximum power density (P_max_/A_m_) with the pressure bias. In equilibrium EKEC, it is known that the maximum power follows the scaling law: P_max_ ~ (p_ext_)^2^ [12,13]. However, the results indicated that the maximum power generated in the forward pressure bias began deviating from this scaling law when the nonequilibrium ICP became considerable at lower salt concentrations and higher pressure biases. Then, the reverse-pressure bias had a higher maximum power than the forward pressure bias. Interestingly, it was found that the maximum power for the reverse pressure bias in the specific range of pressure bias was slightly larger than the value predicted by this scaling law under the assumption of equilibrium EKEC. The dependence of the pressure bias on the EKEC conversion efficiency is presented in Figure 9b. The EKEC conversion efficiency (*η*) is defined as the ratio of output electrical power and input mechanical power:

(7) $$\eta =\frac{\left|I\right|\left|V\right|}{\left|Q\right|p_{ext}}$$

The maximum value of *η* in the I-*η* curve is referred to as the maximum conversion efficiency (*η*_max_) of EKEC. As expected, the maximum conversion efficiency in the forward-pressure bias decreased with increasing pressure bias due to the impact of ICP. Notably, the reverse pressure bias revealed a slight increase in the maximum conversion efficiency in the specific range of pressure bias because the maximum power generated in the reverse pressure bias was slightly enhanced by the nonequilibrium ICP, as shown in Figure 9a. The difference between the forward pressure bias and reverse-pressure bias in the maximum conversion efficiency became significant as the pressure bias increased at the salt concentration of c_0_ = 0.1 mM. Consequently, it is concluded that the maximum conversion efficiency in the forward-pressure bias is slightly larger than that in the reverse-pressure bias when the ICP effect is negligible. In contrast, the maximum conversion efficiency in the reverse pressure bias becomes significantly larger than the forward pressure bias due to the strongly nonequilibrium ICP effect at lower salt concentrations and higher pressure biases.

**Figure 10:**
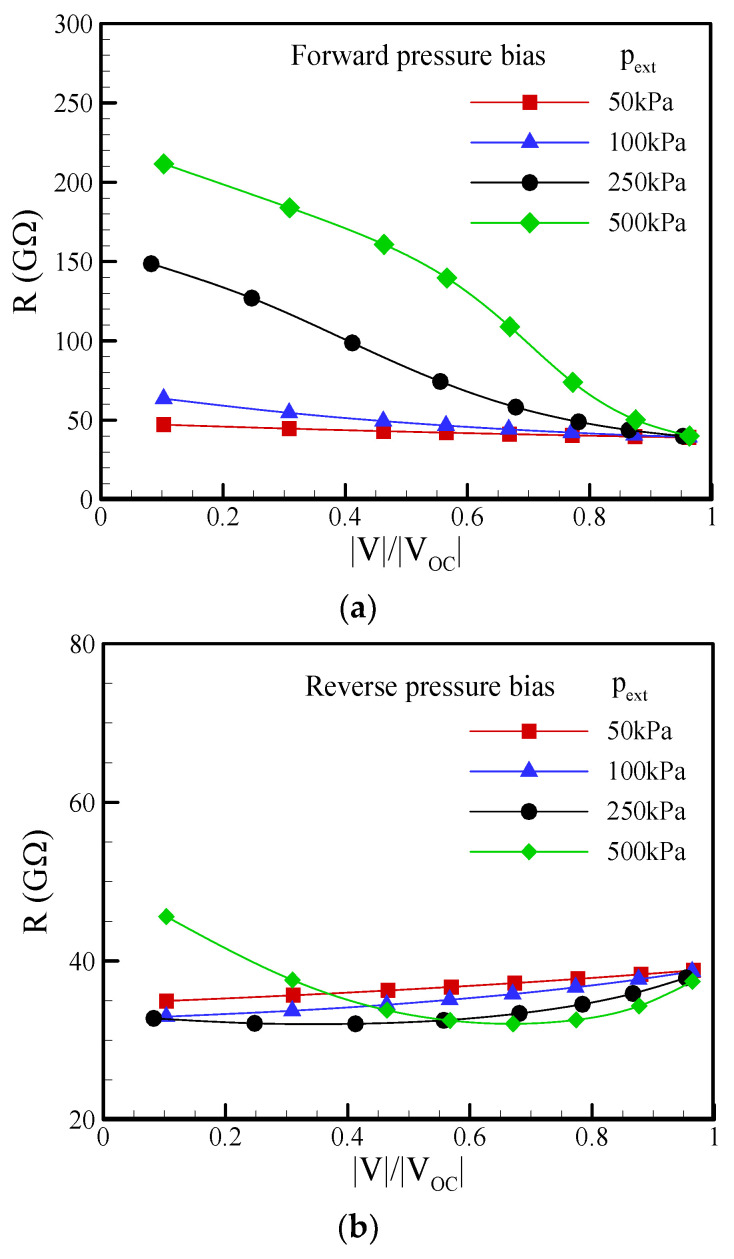
Plots of the variation in electrical resistance (R) with voltage |V| at a salt concentration of c_0_ = 0.1 mM under different pressure biases: (**a**) forward pressure bias; (**b**) reverse pressure bias. Note that *θ* = 1.2°, *σ*_s_ = −5 mC/m^2^, and b = 20 nm.

**Figure 11:**
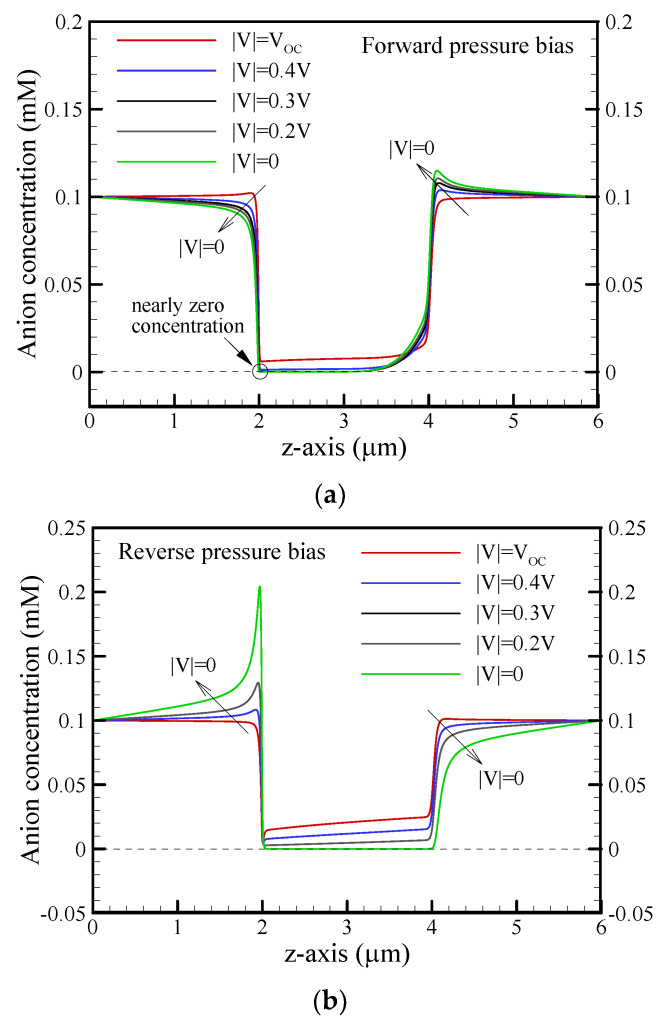
Plots of the anion (Cl^−^) concentration along the centerline of the reservoir and conical nanopore for different voltages of |V| at a salt concentration of c_0_ = 0.1 mM and pressure bias of p_ext_ = 500 kPa. (**a**) Forward pressure bias; (**b**) Reverse pressure bias. Note that *θ* = 1.2°, *σ*_s_ = −5 mC/m^2^, and b = 20 nm.

Why does the conical nanopore exhibit asymmetric behavior in EKEC? The mechanism is mainly attributed to the asymmetric behavior in ICP, including ion depletion and ion enrichment under the forward/reverse pressure bias. In Figure 10, it can clearly be seen that the electrical resistance (R) (the summation of access resistance and pore resistance) varied with the voltage |V| in the nonlinear I-V curves, as presented in Figure 8. In the forward pressure bias, the electrical resistance increased with decreasing voltage |V|, as shown in Figure 10a, because the ion depletion around the tip of the conical nanopore became more significant as the voltage decreased, as shown in Figure 11a. The results indicate that the anion concentration around the tip approached nearly zero with decreasing voltage. Because the electrical resistance is inversely proportional to the ion concentration, the nearly zero anion concentration on the tip side resulted in a higher local electrical resistance and a higher electrical resistance (R), as shown in Figure 8, even though ion enrichment occurred around the base of the nanopore. In other words, the change in electrical resistance was dominated by ion depletion in the forward-pressure bias. The increase in electrical resistance became more significant with increasing pressure bias and decreased the maximum power and conversion efficiency, as shown in Figure 9. In contrast, Figure 10b shows that the electrical resistance in the reverse-pressure bias decreased with decreasing voltage since the ion enrichment around the tip of the conical nanopore became more significant, as seen in Figure 11b. Although ion depletion occurred around the base of the nanopore, the ion-depleted concentration was obviously higher than that in the forward-pressure bias. As a result, the change in electrical resistance was dominated by the ion enrichment, and the decrease in electrical resistance was observed in the reverse-pressure bias except when the nearly zero ion-depleted concentration was induced at a larger pressure bias. In addition, the decrease in electrical resistance became more significant as the pressure bias increased. It enhanced the maximum power and maximum conversion efficiency, as shown in Figure 9, and the EKEC performance increased as the pressure bias increased. However, when the change in electrical resistance was gradually dominated by ion depletion at a larger reverse-pressure bias, the electrical resistance began increasing with decreasing voltage |V|, as seen in Figure 10b. As a result, the decrease in maximum power and maximum conversion efficiency at the larger pressure bias is observed in Figure 9. Consequently, the reverse-pressure bias revealed a lower electrical resistance than the forward-pressure bias and resulted in asymmetrical EKEC characteristics in a conical nanopore.

### 3.4. Effect of the Slip Length, Salt Concentration, Surface Charge Density and Cone Angle on the Maximum Power and Maximum Conversion Efficiency of EKEC

**Figure 12:**
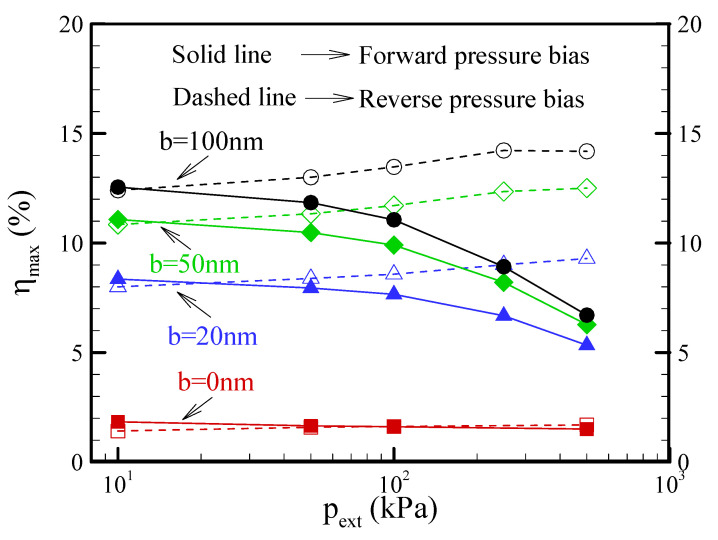
Plot of the maximum conversion efficiency (*η*_max_) as a function of the pressure bias for EKEC in a conical nanopore with different slip lengths of b = 0, 20, 50, and 100 nm at a salt concentration of c_0_ = 0.1 mM. Note that *θ* = 1.2° and *σ*_s_ = −5 mC/m^2^.

Figure 12 reveals the effect of the slip length on the maximum conversion efficiency for EKEC in conical nanopores at a salt concentration of c_0_ = 0.1 mM. As expected, the maximum conversion efficiency was greatly improved by increasing the slip length. Likewise, it can be seen that the maximum conversion efficiency in the reverse-pressure bias was enhanced by the ICP effect as the pressure bias was increased, particularly for a larger slip length. Notably, the access resistance of the nanopore became very considerable in an ultrashort nanopore, and caused a decrease in the power generation and conversion efficiency [16]. Accordingly, the value of slip-enhanced conversion efficiency for EKEC in a finite-length conical nanopore reported in this study is smaller than the predicted value for an infinite-length nanopore [13].

**Figure 13:**
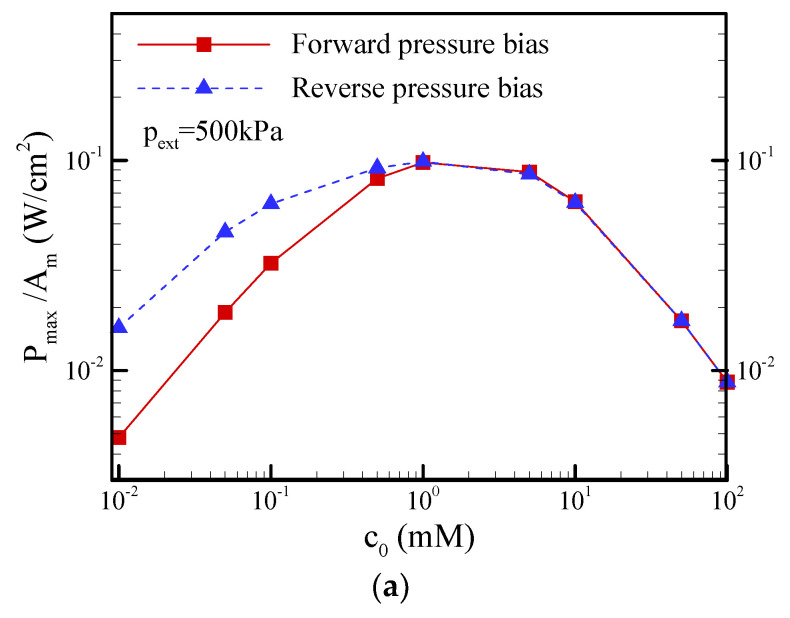

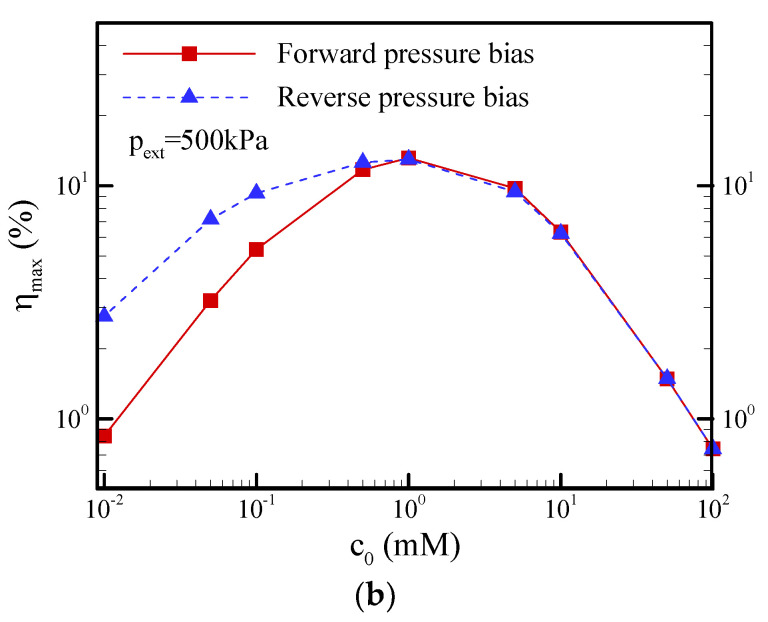
Plots of the (**a**) maximum power density (P_max_/A_m_) and (**b**) maximum conversion efficiency (*η*_max_) as a function of the salt concentration under a pressure bias of p_ext_ = 500 kPa. Note that *θ* = 1.2°, *σ*_s_ = −5 mC/m^2^, and b = 20 nm.

Figure 13 shows the variations in the maximum power density and maximum conversion efficiency with the salt concentration under a pressure bias of 500 kPa. It can be seen that the maximum power density and maximum conversion efficiency both increased with decreasing salt concentration until the salt concentration of c_0_ = 1 mM in both the forward pressure bias or reverse pressure bias and then decreased as the salt concentration was further decreased. This decrease is due to the considerable access resistance and ICP effects at the lower salt concentration of less than c_0_ = 1 mM. Above the value of c_0_ = 1 mM, there was no obvious distinction between the forward pressure bias and the reverse pressure bias. When the salt concentration was lower than c_0_ = 1 mM, asymmetrical behavior in EKEC was observed. As discussed in the last section, the reverse pressure bias revealed a higher EKEC performance than the forward pressure bias. The discrepancy due to the asymmetric behavior increased with decreasing salt concentration.

**Figure 14:**
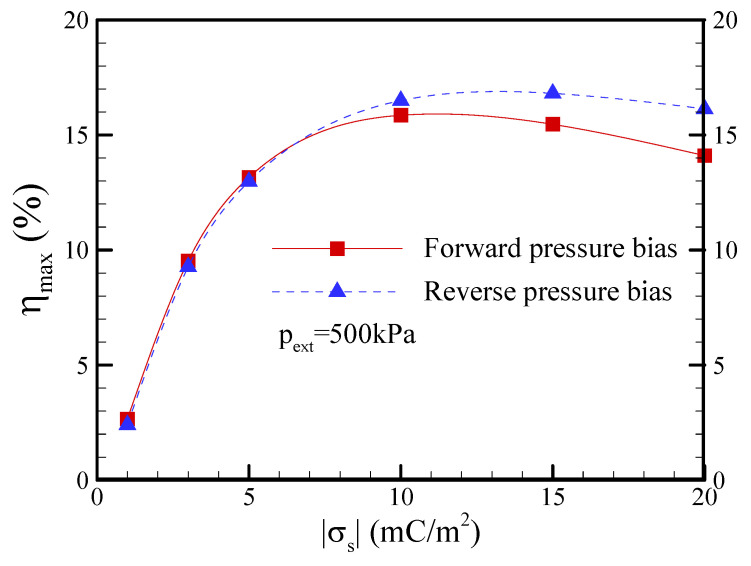
Plots of the maximum conversion efficiency (*η*_max_) as a function of the surface charge density under the conditions of c_0_ = 1 mM and p_ext_ = 500 kPa. Note that *θ* = 1.2° and b = 20 nm.

**Figure 15:**
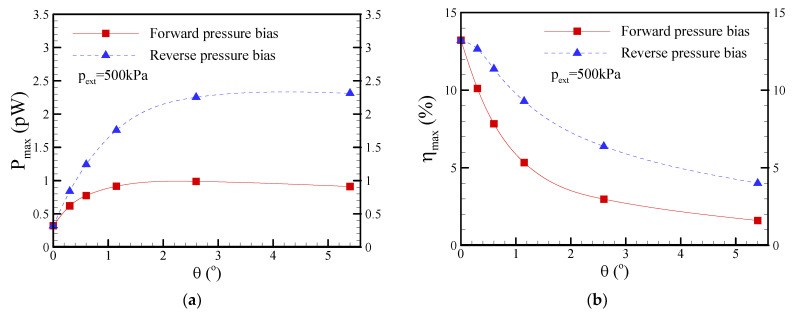
Plots of the (**a**) maximum power (P_max_) and (**b**) maximum conversion efficiency (*η*_max_) as a function of the half-cone angle (*θ*) under the conditions of c_0_ = 0.1 mM and p_ext_ = 500 kPa. Note that *σ*_s_ = −5 mC/m^2^ and b = 20 nm. The half-cone angle was adjusted by changing the base radius a_b_.

Figure 14 presents the effect of the surface charge density on the maximum conversion efficiency at a salt concentration of c_0_ = 1 mM. The maximum conversion efficiency dramatically increased in the low-surface-charge density regime and slightly decreased in the high-surface-charge density regime. The asymmetric behavior in EKEC became more significant in the higher-surface-charge density regime even though EDL overlap was not very significant (i.e., a_t_/*λ*_D_ = 1.0 and a_b_/*λ*_D_ = 5). This is because the surface conductance contributed from the higher-surface-charge density dominated the ionic conductance of the nanopore (i.e., the Dukhin number is larger than unity [48]) and resulted in a more significant ICP effect at a higher salt concentration of c_0_ = 1 mM [36]. Note that the Dukhin number represents the ratio of surface conductivity to bulk conductivity. It was suggested that the Dukhin number is more appropriate than the dimensionless Debye-Hückel parameter to evaluate the occurrence of ICP. A larger Du value implies that the nanopore has a higher ion selectivity and thus easily results in the occurrence of ICP. Figure 15 reveals the effect of the cone angle on the maximum power and maximum conversion efficiency at a salt concentration of c_0_ = 0.1 mM. In Figure 15a, it is apparent that the maximum power increased as the cone angle was increased below the half-cone angle of *θ* = 1.2° due to the decrease in the hydraulic resistance of nanopore, i.e., the scaling law of the flow rate |Q|~a^4^ indicates the higher flow rate produced in a larger base radius of nanopore (cone angle) and drives the higher streaming current/potential and electrical power. Note that the EDL almost overlapped below the half-cone angle of *θ* = 1.2° dimensionless since the dimensionless Debye-Hückel parameter of a/*λ*_D_ was smaller than 1.5. However, the maximum power reached a plateau above the half-cone angle of *θ* = 1.2° since the excess counterions within the nonelectroneutral EDL could not be dragged effectively by the lower flow velocity near the pore wall when the dimensionless Debye-Hückel parameter of a/*λ*_D_ became large. On the other hand, the maximum conversion efficiency decreased with increasing cone angle, as shown in Figure 15b, because the increase in the input mechanical power (|Q|p_ext_~a^4^) shown in Equation (7) was larger than the output electrical power as the cone angle increased. In addition, asymmetric behavior in the maximum power and maximum conversion efficiency was observed, and the reverse pressure bias revealed a higher EKEC performance.

## 4. Conclusions

In this study, the characteristics of EKEC in conical nanopores with slip effects were investigated numerically. The results indicated that the EKEC exhibits nonlinear behavior once the nonequilibrium ICP phenomenon occurs. Transport properties such as the streaming current/potential and flow rate revealed rectification phenomena under forward pressure bias and reverse pressure bias. Notably, the direction of rectification for EKEC was opposite to that of the well-known ICR phenomenon observed in conical nanopores. From the nonlinear characteristics of the I-V curves, it was found that ion depletion always dominated the change in electrical resistance under a forward pressure bias and resulted in an increase in electrical resistance. In contrast, a decrease in electrical resistance was observed under a reverse pressure bias when the change in electrical resistance was dominated by ion enrichment under certain conditions. As a result, the EKEC under the reverse pressure bias revealed a higher maximum power and maximum conversion efficiency than the forward pressure bias. In other words, the EKEC exhibited asymmetrical behavior in a conical nanopore. Notably, the maximum power and conversion efficiency both were enhanced under a reverse pressure bias since the ion enrichment decreased the electrical resistance. Furthermore, it was found that the asymmetric EKEC characteristics became more significant with increasing slip length, surface charge density, cone angle, and pressure bias, particularly at lower salt concentrations. It is believed that these findings will contribute to the development of EKEC based on engineered membranes with asymmetric nanopores, e.g., ion-tracked PET membranes.

## Figures and Tables

**Table 1:** Summary of the boundary conditions used in the computation.

Surface	Ion Concentration Field	Electric Field	Flow Field
AB, CD	zero normal flux $n\cdot j_{i}=0$	zero charge n·∇ϕ=0	u·n=0 $n\cdot \left(\nabla u+{\left(\nabla u\right)}^{T}\right)=0$
BG, GH, CH	zero normal flux $n\cdot j_{i}=0$	surface charge density $-n\cdot \varepsilon_{f}\varepsilon_{0}\nabla \phi =\sigma_{s}$	no penetration u·n=0 Navier slip velocity $u=bn\cdot \left(\nabla u+{\left(\nabla u\right)}^{T}\right)$
AE	bulk concentration $c=c_{\infty }$	ϕ=V ϕ=0 for short circuit n·∇ϕ=0 for open circuit	$p=p_{\mathrm{ext}}$ for forward bias p=0 for reverse bias
DF	bulk concentration $c=c_{\infty }$	grounded ϕ=0	p=0 for forward bias $p=p_{\mathrm{ext}}$ for reverse bias
EF	axial symmetry	axial symmetry	axial symmetry

## Data Availability

Data will be provided via requests to the corresponding author.
